# Comments on Case Report: A fatal case of *Aspergillus felis* infection in an immunocompetent host

**DOI:** 10.1099/acmi.0.000561.v3

**Published:** 2023-04-03

**Authors:** Vanessa R. Barrs

**Affiliations:** ^1^​ Department of Veterinary Clinical Sciences and Centre for Animal Health and Welfare, Jockey Club College of Veterinary Medicine and Life Sciences, City University of Hong Kong, Hong Kong, SAR, PR China

**Keywords:** *Aspergillus viridinutans* complex, veterinary mycology, aspergillosis

## Full-Text

I read with sadness the report of an immunocompetent 18-year-old man with fatal fungal rhinosinusitis (FRS) and cerebral abscess caused by *Aspergillus felis*.

The parallels between this case and infections reported in immunocompetent domestic cats are striking. *A. felis* was described a decade ago after its isolation from pet cats in Australia with invasive fungal rhinosinusitis (FRS) characterized by sinonasal and sino-orbital involvement [1, 2]. As in this human case report, cats usually succumb once there is central nervous system (CNS) involvement, despite aggressive treatment with targeted antifungals [3].

*A. felis* is an environmental saprophyte that has been isolated from diverse environments on most continents [4]. Parkes-Smith *et al*. state that there are six pathogenic species within the *Aspergillus viridinutans* complex: *A. udagawae*, *A. felis*, *A. pseudofelis*, *A. parafelis*, *A. pseudoviridinutans* and *A. wyomingensis*. However, the complex has undergone extensive revision after sequencing of clinical and environmental strains from six continents [5]. It is now recognized that *A. felis*, *A. parafelis* and *A. pseudofelis* are, in fact, one species (*A. felis*). Ten species make up the *A. viridinutans* complex, two of which have only been isolated from Australia – *A. viridinutans* [6] and *A. frankstonensis* [7]. The former was isolated from rabbit dung in Frankston in 1954, and the latter from soil and air by former PhD student in my laboratory, Dr Jess Talbot, after we drove to Frankston, on a whim in 2015, to do some environmental sampling during the ISHAM Congress in Melbourne.

I have wondered why it is that apparently immunocompetent cats develop invasive FRS. Over-representation of Persian-related cat breeds raises the possibility of an uncharacterized genetic defect in fungal immunity or of impaired sinus drainage due to foreshortening of the skull (brachycephaly). In favour of the former, these same breeds of cats are also susceptible to other invasive fungal infections (IFIs), such as dermatophytic pseudomycetoma. There has been very limited veterinary research to investigate putative immunological defects. The findings from one study did not support a role for single-nucleotide polymorphisms (SNPs) in the coding regions of pattern recognition receptor genes, specifically Toll-like receptor (TLR) genes 1, 2 and 4, in the pathogenesis of fungal rhinosinusitis caused by *Aspergillus* species in cats [8] .

Regardless, invasive FRS also occurs in immunocompetent crossbred cats with no known underlying risk factors. An analogous condition, that of chronic granulomatous invasive fungal sinusitis, caused by *Aspergillus flavus*, has been reported for decades in immunocompetent or mildly immunosuppressed people from Sudan, India, Pakistan and Saudia Arabia [9]. The environmental burdens of *A. flavus* conidia are high in these regions [10]. Exposure to high environmental spore burdens may be the most logical explanation for the development of invasive FRS in some immunocompetent cats.

A role for species-specific fungal virulence factors in the development of invasive FRS in immunocompetent individuals must also be considered. In experimental models the *A. felis* type strain (isolated from a cat with sino-orbital aspergillosis) showed significantly higher virulence in *Galleria mellonella* larvae than *Aspergillus fumigatus*. In immunosuppressed BALB/c mice, *A. felis* showed slightly higher virulence than *A. fumigatus*. However, in a chronic granulomatous disease murine model, inoculation with *A. fumigatus*, unlike *A. felis,* was uniformly fatal, demonstrating decreased virulence in hosts deficient in production of reactive oxygen species [11]. Another study investigated changes on computed tomography of the head in immunocompetent cats with invasive and non-invasive FRS caused by any *Aspergillus* species, where the infecting isolate had been identified by PCR and sequencing of the internal transcribed spacer (ITS) regions (ITS1–5.8S-ITS2), partial β-tubulin and/or partial calmodulin genes [12]. There was a strong association between the infecting species and anatomical site of aspergillosis, with infections caused by *A. fumigatus* confined to the sino-nasal cavity, while cryptic species infections (mostly caused by *A. felis* and *A. udagawae*) were associated with invasive sino-orbital disease, including orbital and paranasal soft tissue involvement and lysis of the orbital bone.

A further consideration is that *A. viridinutans* complex members are heterothallic but, unlike *A. fumigatus*, readily produce teleomorphs [5]. Whether there is any difference in the ability of asexual (conidia) or larger sexual spores (ascospores) to induce invasive infections, and the comparative localization of these inhaled bioaerosols, has not been investigated.

The antifungal susceptibility profile of the *A. felis* isolate reported by Parkes-Smith *et al*. is typical of most *A. viridinutans* complex members, i.e. low MICs/MECs of posaconazole and echinocandins, respectively, elevated MICs of voriconazole and itraconazole and variable MICs of amphotericin B. The combination of posaconazole and liposomal amphotericin B used in the report was also used to cure a cat with FRS caused by *A. felis* [1]. More recently, two cats with sino-orbital aspergillosis caused by *A. udagawae* and *A. felis* were cured with combined therapy including posaconazole, caspofungin and terbinafine and remained asymptomatic more than 2 years after presentation [13]. A human patient with X-linked chronic granulomatous disease who developed severe vertebral osteomyelitis caused by *A. udagawae* was treated successfully using posaconazole and caspofungin [14].

Finally, a shout-out to the One Health approach of Australian microbiologists and to the International Society of Human and Animal Mycoses (ISHAM), where sharing of comparative human and animal data has long been actively encouraged. Together, we can learn more.
